# Association between use of novel glucose-lowering drugs and COVID-19 hospitalization and death in patients with type 2 diabetes: a nationwide registry analysis

**DOI:** 10.1093/ehjcvp/pvac044

**Published:** 2022-08-13

**Authors:** Giulia Ferrannini, Lars H Lund, Lina Benson, Manfredi Rizzo, Wael Almahmeed, Giuseppe M C Rosano, Gianluigi Savarese, Francesco Cosentino

**Affiliations:** Division of Cardiology, Department of Medicine, Karolinska Institute, Stockholm, Sweden; Division of Cardiology, Department of Medicine, Karolinska Institute, Stockholm, Sweden; Heart and Vascular Theme, Karolinska University Hospital, Stockholm, Sweden; Division of Cardiology, Department of Medicine, Karolinska Institute, Stockholm, Sweden; School of Medicine, ProMISE Department, University of Palermo, Palermo, Italy; Heart and Vascular Institute, Cleveland Clinic Abu Dhabi, Abu Dhabi, UAE; Centre for Clinical and Basic Research, IRCCS San Raffaele Roma, Rome, Italy; Division of Cardiology, Department of Medicine, Karolinska Institute, Stockholm, Sweden; Heart and Vascular Theme, Karolinska University Hospital, Stockholm, Sweden; Division of Cardiology, Department of Medicine, Karolinska Institute, Stockholm, Sweden; Heart and Vascular Theme, Karolinska University Hospital, Stockholm, Sweden

**Keywords:** COVID-19, Sodium-glucose cotransporter 2 inhibitors, Glucagon-like peptide-1 receptor agonists, Dipeptidyl peptidase-4 inhibitors (DPP-4i), Hospitalization, Mortality

## Abstract

**Aims:**

Type 2 diabetes (T2DM) in patients with coronavirus disease-19 (COVID-19) is associated with a worse prognosis. We separately investigated the associations between the use of sodium-glucose cotransporter 2 inhibitors (SGLT2i), glucagon-like peptide-1 receptor agonists (GLP-1 RA), and dipeptidyl peptidase-4 inhibitors (DPP-4i), and the risk of COVID-19 hospitalization and death.

**Methods and results:**

Patients with T2DM registered in the Swedish National Patient Registry and alive on 1 February 2020 were included. ‘Incident severe COVID-19’ was defined as the first hospitalization and/or death from COVID-19. A modified Poisson regression approach was applied to a 1:1 propensity score-matched population receiving vs. not receiving SGLT2i, GLP-1 RA, and DPP-4i to analyse the associations between their use and (I) incident severe COVID-19 and (II) risk of 30-day mortality in patients hospitalized for COVID-19.

Among 344 413 patients, 39 172 (11%) were treated with SGLT2i, 34 290 (10%) with GLP-1 RA, and 53 044 (15%) with DPP-4i; 9538 (2.8%) had incident severe COVID-19 by 15 May 2021. SGLT2i and DPP-4i were associated with a 10% and 11% higher risk of incident severe COVID-19, respectively, whereas there was no association for GLP-1 RA. DPP-4i was also associated with a 10% higher 30-day mortality in patients hospitalized for COVID-19, whereas there was no association for SGLT2i and GLP-1 RA.

**Conclusion:**

SGLT2i and DPP-4i use were associated with a higher risk of incident severe COVID-19. DPP-4i use was associated with higher 30-day mortality in patients with COVID-19, whereas SGLT2i use was not. No increased risk for any outcome was observed with GLP-1 RA.

## Introduction

The current coronavirus disease 2019 (COVID-19) pandemic, due to the severe acute respiratory syndrome coronavirus-2 (SARS-CoV-2), is an ongoing challenge.^1^Type 2 diabetes mellitus (T2DM) has been reported as one of the most frequent co-morbidities associated with severe COVID-19, conferring a two-fold higher relative risk of severe COVID-19 requiring intensive care unit and in-hospital death.^2^ Possible mechanisms behind higher morbidity and mortality with COVID-19 in patients with vs. without T2DM are systemic inflammation, immunodeficit, and hypercoagulability.^3,4^The cytokine storm in severe COVID-19 involves elevated levels of serum C-reactive protein, interleukin-6 (IL-6), D-dimer, and ferritin, which are also observed in the chronic inflammation associated with hyperglycaemia.^5^Angiotensin converting enzyme 2 (ACE2) and dipeptidyl peptidase-4 (DPP-4) are two coronavirus receptor proteins that also have a role in glucose homeostasis regulation.^6,7^ Finally, observational studies suggested that anti-inflammatory agents used in severe COVID-19 pneumonia, e.g. anti-IL-6 agents, might be less effective in the presence of hyperglycaemia.^8^

Novel glucose-lowering medications may reduce adverse COVID-19 outcomes because of their anti-inflammatory properties, but the potential role of different pharmacological classes in adverse COVID-19 outcomes has not been systematically investigated.^5^ DPP-4 inhibitors (DPP-4i) have been suggested to have a beneficial role in T2DM patients hospitalized for COVID-19.^5,9^ Sodium-glucose cotransporter-2 inhibitors (SGLT2i) and glucagon-like peptide-1 receptor agonists (GLP-1 RA) have several anti-inflammatory properties, which might also be linked with better outcomes.^5,10^ Conversely, safety concerns have been raised for SGLT2i and GLP-1 RA since they increase ACE2 expression, which mediates SARS-CoV-2 binding to the cells.^11^ However, RAAS inhibitor drugs, which also increase ACE2 expression, do not appear to be associated with an increased risk of incident COVID-19 or worse outcomes in prevalent COVID-19.^12^

The aim of the current study was to separately investigate the association between SGLT2i, GLP-1 RA, and DPP-4i use with (I) incident hospitalization/death for COVID-19 and (II) mortality in patients with COVID-19 in a nationwide cohort of T2DM patients in Sweden.

## Methods

### Data sources

The analyses were performed using the Swedish National Patient Registry (NPR) linked through the personal identification number to the Cause of Death Registry, the Dispensed Drug Registry, and Statistics Sweden.^13^ The NPR, the Cause of Death Registry, and the Dispensed Drug Registry are administered by the Swedish Board of Health and Welfare (www.socialstyrelsen.se), which collects International Classification of Diseases (ICD-10) diagnoses from all residents in Sweden, at hospitalizations as well as at outpatient non-primary care clinics. The Dispensed Drug Registry contains data for all dispensed prescriptions since 2005. Statistics Sweden collects socioeconomic data of Swedish residents.

### Study population and outcomes

Adult patients with a diagnosis of T2DM in the NPR after 1997 (when ICD-10 was implemented) and who were alive on 1 February 2020 were included in the analyses. Additional exclusion criteria are reported in Supplementary material online, *Table S1*.

Index date was 1 February 2020 (the first COVID-19 case in Sweden was registered at the end of January 2020). End of follow-up was 15 May 2021.

Outcomes were incident severe COVID-19 in the overall study population and 30-day all-cause mortality in patients with COVID-19. Incident severe COVID-19 was defined as the first occurrence of hospitalization with confirmed COVID-19 as the main diagnosis in the NPR or confirmed COVID-19 as the underlying cause of death in the Cause of Death Registry. In patients hospitalized for COVID-19, subsequent hospitalizations for hypoglycaemia and diabetic ketoacidosis (DKA) were also investigated, with patients censored at death or at end of follow-up.

The percentage of patients still on treatment with the different study drugs was calculated based on those with at least one prescription within 5 months after an incident severe COVID-19.

Detailed definitions for co-morbidities, COVID-19 disease, and treatments are available in Supplementary material online, *Table S2*.

### Statistical analysis

Baseline characteristics of patients receiving vs. not receiving SGLT2i, GLP-1 RA, and DPP-4i, separately, were reported as frequencies (percentages) for categorical variables and as medians (interquartile range––IQR) for continuous variables. Differences were evaluated by standardized mean differences (SMD), where a value < 0.1 was considered as non-significant. There were limited missing data from the following variables from Statistics Sweden: country of birth, income, education level, family type (living alone or not), and living in the region of Stockholm or not. Patients with missing data are excluded from all analyses (Supplementary material online, *Table S1*).

Separate analyses were performed for the three investigated drug classes. In the whole study population, the association between treatment and incident severe COVID-19 was evaluated. In a subset of patients hospitalized for COVID-19 and 30-days follow-up available, the association between the treatment and 30-day all-cause mortality was assessed. The associations were investigated by a modified Poisson regression approach,^14^ i.e. using generalized estimating equations models with a Poisson distribution and a robust error variance. Adjustment for covariates was performed by propensity score (PS) matching, where the PS for the treatment of interest was estimated for each patient by a logistic regression model including the variables indicated with * in Supplementary material online, *Table S3* as covariates, and where age was modelled using cubic splines with four degrees of freedom. A 1:1 matching without replacement, where the PS was allowed to differ by 0.01 or less, was thereafter performed. The ability of the PS-matching to balance the baseline characteristics was assessed by SMD. A 1:1 PS-matching was deemed the best option when the balance between groups and the number of patients retained in the analysis is considered. The matched pairs were incorporated into the model using an exchangeable correlation structure.

Consistency analyses were performed (1) in the overall (unmatched) population, adjusting for the individual variables indicated with * in Supplementary material online, *Table S3* rather than matching by PS; and (2) for the analysis with COVID-19 as an outcome, using a sub-distributional hazards model (Fine–Gray model) for time to incident severe COVID-19 where non-COVID-19 death was treated as a competing event.

The associations between each treatment and the outcomes in predefined subgroups were investigated by including an interaction term in the model. One considered subgroup was the Stockholm region, since the greatest number of cases was registered there. All analyses were performed using R version 4.0.2.

### Ethics

Patient consent is not required for registration in the national administrative registries. The current analysis was approved by the Swedish Ethics Review Authority and was conducted in accordance with the Declaration of Helsinki.

## Results

Of 365 537 patients with a diagnosis of T2DM recorded in the NPR between 1997 and 1 February 2020, 344 413 were included in our analysis after applying the exclusion criteria (Supplementary material online, *Table S1*). The median age (IQR) of the study population was 72 (62–79), 42.4% were women; 39 172 (11.4%) were treated with SGLT2i, 34 290 (10%) with GLP-1 RA, and 53 044 (15.4%) with DPP-4i.

### Baseline characteristics

The baseline characteristics of patients receiving vs. non-receiving SGLT2i, GLP-1 RA, and DPP-4i are reported in *Table** 1* and in Supplementary material online, *Table S3*.

**Table 1:** Baseline characteristics of patients with type 2 diabetes according to the use of SGLT2i, GLP-1 RA, and DPP-4i

VARIABLE	SGLT2i no	SGLT2i yes	SMD	GLP1-RA no	GLP1-RA yes	SMD	DPP-4i no	DPP-4i yes	SMD
Age	72.0[63.0, 79.0]	66.0[59.0, 73.0]	0.433	72.0[63.0, 79.0]	66.0[57.0, 73.0]	0.502	71.0[62.0, 78.0]	73.0[65.0, 79.0]	0.155
Male sex	172 002 (56.3)	26 461 (67.6)	0.232	178 181 (57.5)	20 282 (59.1)	0.034	167 018 (57.3)	31 445 (59.3)	0.040
Main cardiovascular co-morbidities									
Atrial fibrillation	55 315 (18.1)	5765 (14.7)	0.092	56 307 (18.2)	4773 (13.9)	0.116	51 080 (17.5)	10 000 (18.9)	0.034
Heart failure	44 595 (14.6)	5286 (13.5)	0.032	45 462 (14.7)	4419 (12.9)	0.051	41 379 (14.2)	8502 (16.0)	0.051
Hypertension	214 204 (70.2)	27 177 (69.4)	0.017	216 942 (70.0)	24 439 (71.3)	0.029	202 744 (69.6)	38 637 (72.8)	0.072
Ischaemic heart disease	80 260 (26.3)	13 218 (33.7)	0.163	84 600 (27.3)	8878 (25.9)	0.031	78 691 (27.0)	14 787 (27.9)	0.019
Previous stroke/TIA	50 345 (16.5)	5020 (12.8)	0.104	51 456 (16.6)	3909 (11.4)	0.150	46 668 (16.0)	8697 (16.4)	0.010
Pharmacological therapy									
Anticoagulant	55 968 (18.3)	6132 (15.7)	0.071	56 868 (18.3)	5232 (15.3)	0.082	51 876 (17.8)	10 224 (19.3)	0.038
Antiplatlet	100 067 (32.8)	15 653 (40.0)	0.150	104 173 (33.6)	11 547 (33.7)	0.002	96 678 (33.2)	19 042 (35.9)	0.057
Beta-blockers	138 010 (45.2)	19 816 (50.6)	0.108	141 082 (45.5)	16 744 (48.8)	0.067	130 696 (44.9)	27 130 (51.1)	0.126
Lipid-lowering	185 554 (60.8)	30 415 (77.6)	0.371	190 623 (61.5)	25 346 (73.9)	0.269	177 739 (61.0)	38 230 (72.1)	0.236
MRA	21 172 (6.9)	3423 (8.7)	0.067	21 620 (7.0)	2975 (8.7)	0.064	20 479 (7.0)	4116 (7.8)	0.028
RASi/ARNi	189 049 (61.9)	28 361 (72.4)	0.224	192 892 (62.2)	24 518 (71.5)	0.199	180 954 (62.1)	36 456 (68.7)	0.140

Categorical variables are presented with *n* (%) and continuous variables with median [q1–q3].

Abbreviations. DPP-4i, dipeptidyl peptidase-4 inhibitors; GLP-1 RA, glucagon-like peptide-1 receptor agonists; MRA, mineralocorticoid receptor antagonists; RASi/ARNi, renin–angiotensin system inhibitors/angiotensin receptor-neprilysin inhibitors; SGLT2i, sodium-glucose cotransporter 2 inhibitors; SMD, stardardised mean difference; TIA, transitory ischaemic attack.

SGLT2i users vs. non-users had a higher prevalence of obesity and ischaemic heart disease, with more frequent prior coronary revascularizations. SGLT2i users were more likely to receive antiplatelet therapy, renin–angiotensin system inhibitors/angiotensin receptor-neprilysin inhibitors (RASi/ARNi), beta-blockers, and lipid-lowering drugs compared with non-users. Moreover, patients receiving SGLT2i were more often treated with other glucose-lowering agents, including oral antidiabetics, insulin, GLP-1 RA, and DPP-4i.

GLP-1 RA users vs. non-users were younger, with higher education levels and income. The prevalence of the analysed co-morbidities was similar in both groups except for history of stroke, atrial fibrillation, previous bleeding events, history of cancer in the last 3 years, dementia and previous stroke being more prevalent among non-users, and obesity being more common among users. GLP-1 RA users were more often treated with RASi/ARNi, lipid-lowering drugs, insulin, metformin, and SGLT2i but not with other oral antidiabetics.

DPP-4i users vs. non-users were older, with a higher prevalence of renal disease, but the other co-morbidities did not substantially differ. RASi/ARNi, beta-blockers, calcium channel blockers, lipid-lowering drugs, diuretics, and oral glucose-lowering agents were all prescribed in higher proportions in users than in non-users.

Baseline characteristics of the subset of patients hospitalized for COVID-19 according to the use of the three different drug classes are shown in Supplementary material online, *Table S4*.

### Association between SGLT2i, GLP-1 RA, or DPP-4i use and risk of incident severe COVID-19 (*Table**2*)

Of the 344 413 patients included in our analysis, 9538 (2.8%) had incident severe COVID-19; among them, 963 (10.1%) were taking SGLT2i, 907 (9.5%) were taking GLP-1 RA, and 1639 (17.2%) were taking DPP-4i.

**Table 2:** Association between SGLT2i, GLP1-RA or DPP-4i use, and risk of incident COVID-19 outcome, i.e. presence of a hospitalization with confirmed COVID-19 as main diagnosis in the National Patient Registry or as confirmed COVID-19 as underlying cause of death in the Cause of Death Registry

Model	SGLT2i no	SGLT2i yes	*P*-value	GLP1-RA no	GLP1-RA yes	*P*-value	DPP-4i no	DPP-4i yes	*P*-value
Matched, *n* (%) event	864 (2.2)	962 (2.5)		862 (2.5)	906 (2.7)		1485 (2.8)	1639 (3.1)	
RR (95% CI)	ref	1.11 (1.02–1.22)	0.020	ref	1.05 (0.96–1.15)	0.289	ref	1.10 (1.03–1.18)	0.005
Competing risk HR (95% CI)	ref	1.11 (1.02–1.22)	0.021	ref	1.05 (0.96–1.15)	0.290	ref	1.11 (1.03–1.19)	0.005
All patients, *n* (%) event	8575 (2.8)	963 (2.5)		8631 (2.8)	907 (2.7)		7899 (2.7)	1639 (3.1)	
Crude RR (95% CI)	ref	0.88 (0.82–0.93)	<0.001	ref	0.95 (0.89–1.02)	0.140	ref	1.14 (1.08–1.20)	<0.001
Adjusted RR (95% CI)	ref	1.09 (1.02–1.16)	0.017	ref	1.10 (1.02–1.18)	0.010	ref	1.15 (1.09–1.22)	<0.001

**Table 3:** Association between SGLT2i, GLP1-RA, and DPP-4i use and risk of all-cause death within 30 days in patients with COVID-19

Model	SGLT2i no	SGLT2i yes	*P*-value	GLP1-RA no	GLP1-RA yes	*P*-value	DPP-4i no	DPP-4i yes	*P*-value
Matched, *n* (%) event	146 (16.9)	152 (17.6)		169 (20.8)	149 (18.3)		489 (31.7)	541 (35.1)	
RR (95% CI)	ref	1.04 (0.85–1.27)	0.694	ref	0.88 (0.73–1.07)	0.201	ref	1.11 (1.00–1.22)	0.046
All patients, *n* (%) event	2823 (34.7)	152 (17.3)		2823 (34.5)	152 (18.2)		2432 (32.6)	543 (35.1)	
Crude RR (95% CI)	ref	0.50 (0.43–0.58)	<0.001	ref	0.53 (0.46–0.61)	<0.001	ref	1.08 (1.00–1.16)	0.059
Adjusted RR (95% CI)	ref	0.91 (0.79–1.05)	0.183	ref	0.91 (0.79–1.04)	0.155	ref	1.05 (0.98–1.12)	0.202

**Figure 1:**
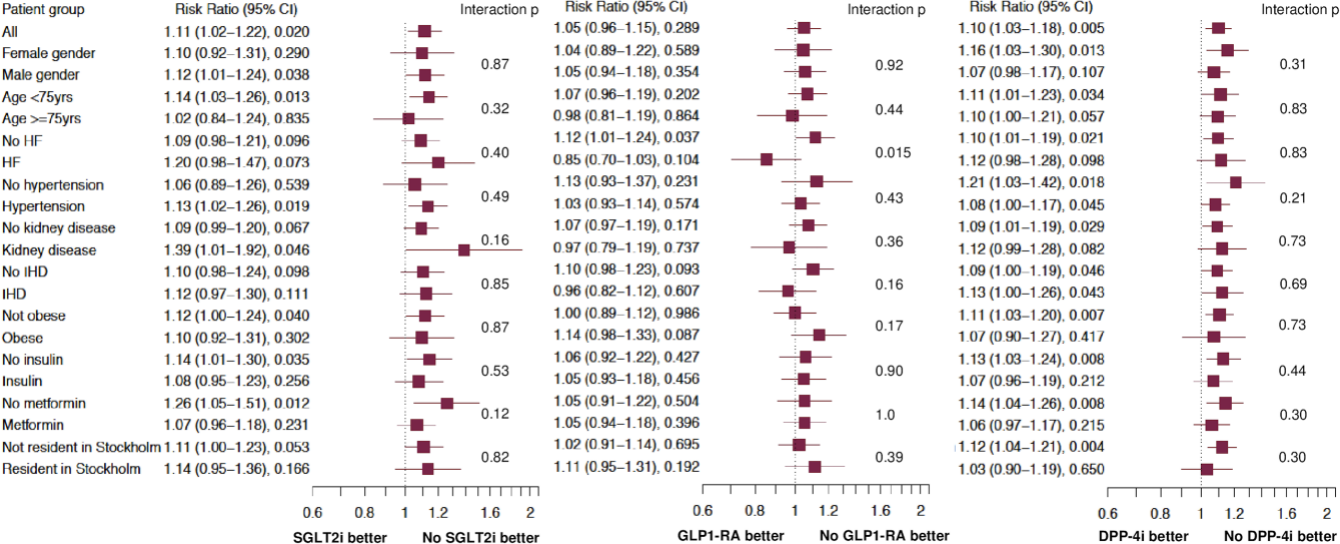
Forest plots of the association between SGLT2i, GLP-1 RA, and DPP-4i and incident severe COVID-19 in the whole propensity-score matched cohort and in relevant subgroups. HF, heart failure; IHD, ischaemic heart disease; SGLT2i, sodium-glucose cotransporter 2 inhibitors; GLP-1 RA, glucagon-like peptide-1 receptor agonists; DPP-4i, dipeptidyl peptidase-4 inhibitors.

The risk of incident severe COVID-19 was significantly higher in SGLT2i users vs. non-users, with a risk ratio (RR) [95% confidence interval (CI)] of 1.11 (1.02–1.22). The consistency analyses confirmed this significant difference, and results were similar across all investigated subgroups (*Figure** 1*).

The use of GLP-1 RA was not significantly associated with the risk of incident severe COVID-19 [RR (95% CI): 1.05 (0.96–1.15)], which was confirmed by the competing risk analysis. However, in the consistency analysis in the unmatched population, where adjustments were performed according to individual covariates, GLP-1 RA use was associated with a higher risk of incident severe COVID-19 (RR [95% CI]: 1.10 [1.02–1.18]). Among the investigated subgroups, GLP-1 RA treatment was associated with a higher risk of incident severe COVID-19 in the subset without heart failure [RR (95% CI): 1.12 (1.01–1.24); *P*-value for interaction 0.015]. Results were consistent in the other subgroups (*Figure**1*).

The use of DPP-4i was associated with a higher risk of incident severe COVID-19 [RR (95% CI): 1.10 (1.03–1.18)], with similar results in the consistency analyses and in all subgroups (*Figure**1*).

### Outcomes in patients hospitalized for COVID-19 (*Table 3*)

Overall, 2975 (31%) deaths occurred in the 9538 hospitalized patients with COVID-19 as a primary diagnosis, with 2145 deaths (72%) having COVID-19 as the underlying cause of death. In the PS-adjusted analyses, there was no significant difference in the risk of 30-day all-cause death in patients receiving vs. not receiving SGLT2i, with a RR (95% CI) of 1.04 (0.85–1.27), and GLP-1 RA with a RR (95% CI) of 0.88 (0.73–1.07), whereas the risk was higher in DPP-4i users vs. non-users, with a RR (95% CI) of 1.11 (1.00–1.22). These results were overall consistent in the subgroups, except for a statistically significant lower risk of 30-day mortality associated with GLP-1 RA use in patients on concomitant metformin therapy [RR (95% CI): 0.62 (0.45–0.85); interaction *P* = 0.003].

Hospitalizations for hypoglycaemia according to the use of the three different drug classes were very low, with no substantial difference between users and non-users, as presented in Supplemental material online, *Table S5*. There were no hospitalizations for DKA.

As regards treatment continuation, 285 (78%) of the patients who were prescribed an SGLT2i at the index date were still prescribed after 5 months; the respective results for GLP1-RA and DPP-4i were 250 (77%) and 400 (78%), respectively.

## Discussion

In this nationwide cohort of patients with T2DM, the use of SGLT2i and of DPP-4i was associated with a higher risk of incident severe COVID-19, defined as hospitalization for or death from COVID-19, whereas GLP-1 RA use was numerically associated with an increased risk but without reaching statistical significance. Among patients hospitalized with COVID-19, the use of DPP-4i, but not of SGLT2i or GLP-1 RA, was associated with higher mortality.

The relationship between the use of glucose-lowering agents and COVID-19-related outcomes is paramount in clinical practice and to policymakers because of the high and growing prevalence of T2DM and subsequent cardiovascular complications, the increasing use of novel glucose-lowering drugs, the higher COVID-19 mortality observed in patients with T2DM,^15^ and the recurring and unpredictable waves of COVID-19 despite effective vaccines.^16^

As regards SGLT2i, clinical practice guidelines recommend the discontinuation in acute illness due to the increased risk of volume depletion and DKA.^17^ We observed that their at-home use was associated with an 11% higher risk of incident severe COVID-19, however, no hospitalizations for DKA were observed. These findings might be explained by higher hospitalization rates for COVID-19 in patients treated vs. non-treated with SGLT2i, with the first being at higher CV risk compared with the latter, and therefore more susceptible to a more severe COVID-19. Indeed, in our population, a larger proportion of patients receiving SGLT2i had established atherosclerotic cardiovascular disease and were on treatment with antiplatelet agents, antihypertensive medications, lipid-lowering drugs, and antidiabetics.^18^ Despite extensive adjustments for these and many other patient characteristics, we were not able to directly assess and therefore adjust for other cardiometabolic risk factors such as glycated haemoglobin (HbA1c), or lipid levels, and thus we cannot rule out that residual confounding, e.g. more severe cardiometabolic disease in SGLT2i users, might explain their 11% higher risk of incident severe COVID-19. However, in patients hospitalized for COVID-19, mortality rates were not higher with SGLT2i. Consistent with these findings, the DARE-19 randomized controlled trial showed no difference between dapagliflozin and placebo in 1250 patients with cardiometabolic risk factors as regards the risk of new or worsened organ dysfunction, death, and recovery.^19^ Nonetheless, dapagliflozin was well tolerated and no new safety signals were identified, in accordance with the present study, where just two hospitalizations for hypoglycaemia were reported for SGLT2i users (vs. seven in non-users), and there was no hospitalization for DKA. Results of the RECOVERY trial (NCT04381936), which aims to assess whether empagliflozin reduces the risk of death, the length of hospital stay, and the need of mechanical ventilation among patients admitted to hospital with COVID-19, will provide further evidence on SGLT2i.

It has been suggested that the pharmacological inhibition of DPP-4 might hinder virus penetration in the target cells, thus conferring a lower risk of incident COVID-19 in DPP-4i users.^20^ However, later preclinical studies showed that the binding sites for DPP-4i do not overlap with those for viral spike proteins of SARS-CoV-2.^21^ Observational data are considerably heterogeneous.^22^ In a large primary care setting in the UK, patients prescribed with SGLT2i had a similar risk of confirmed or clinically suspected COVID-19 compared with patients prescribed with DPP4i.^23^ On the other hand, an observational cohort study on 2.85 million English patients with T2DM reported adjusted hazard ratios for COVID-19-related deaths of 0.94 (0.83–1.07) for GLP-1 RA and 1.07 (1.01–1.13) for DPP-4i inhibitors.^24^

The current practical recommendations do not mandate the discontinuation of incretin-based therapies in patients with COVID-19,^17^ and indeed, most of the patients with incident severe COVID-19 in the present study were prescribed the investigated treatments after discharge. Previous studies on patients with T2DM and confirmed COVID-19 report mixed findings. Meta-analyses reported that the use of DPP-4i(9) and of GLP-1 RA^25^ was associated with decreased COVID-19 mortality. In a study conducted in Italy, treatment with sitagliptin was associated with lower mortality and better clinical outcomes compared with standard-of-care treatment.^26^ Accordingly, a large multinational retrospective cohort study demonstrated that the use of GLP-1 RA and DPP-4i was associated with fewer hospital admissions, respiratory complications, and mortality.^27^ Other studies suggested no associations between incretin-based therapies used in COVID-19 and outcomes: A registry-based Danish study did not show any difference in the risk of adverse outcomes between GLP‐1 RA or DPP‐4i users and SGLT2i users.^28^ In the Spanish SEMI-COVID-19 registry, no significant associations were found between the use of SGLT2i and DPP-4i and the admission to intensive care units, mechanical ventilation, in-hospital death, development of in-hospital complications, and a long-time hospital stay in patients hospitalized for COVID-19.^29^ A PS-matched analysis of the prospective observational study, CORONADO reported no association between DPP-4i use and the composite primary endpoint (tracheal intubation for mechanical ventilation and death within 7 days of admission).^30^ Finally, data from 12 446 SARS-CoV-2-positive adults in the National COVID Cohort Collaborative U.S. study showed that GLP-1 RA and SGLT2i use were associated with lower odds of 60-day mortality compared with DPP-4i use.^31^

GLP-1 RA use was not associated with incident severe COVID-19 and mortality in patients hospitalized for COVID-19 in the main analysis (i.e. PS-matched), whereas in the adjusted model including all patients the risk associated with GLP1-RA use was 10% higher. The association of DPP-4i use with incident severe COVID-19 was significant, consistently in the main analysis and in the multi-adjusted analysis. One possible explanation could be that in our study, DPP-4i users were significantly older compared with non-users and with SGLT2i and GLP-1 RA users, in accordance with national recommendations in Sweden. Age by itself is an independent risk factor for COVID-19 morbidity and mortality and might be accompanied by other co-morbidities and frailty which we could not adjust for. Another possible explanation is that, unlike SGLT2i and most GLP1-RA, DPP-4i does not provide cardiovascular protection in patients with T2DM, and this might affect the prognosis in patients with severe COVID-19, who are particularly burdened by adverse cardiovascular outcomes.^32^ Stronger evidence will be provided by ongoing randomized clinical trials on sitagliptin (SIDIACO-RCT, NCT04365517) and linagliptin (NCT04371978, NCT04341935). However, it must be noted that SGLT2i and GLP-1 RA are very effective in reducing cardiovascular risk, which steadily burdens patients with diabetes at least as much as COVID-19 during the pandemic, thus their benefit justifies their continued use.

The present analysis has several strengths. The inclusion of a large nationwide registry population with full coverage warrants the high generalizability of our findings. The risk of incident severe COVID-19 is addressed in the general population of T2DM patients, providing a large clinical prospective. PS-matched analyses allowed us to adjust for potential known confounders.

Some limitations should be acknowledged. First, the observational nature of this study prevents from assessing causality, i.e. residual confounding and selection bias cannot be ruled out. We made all efforts to address this bias by using different models in consistency analyses, however, the results were not steadily in accordance: In the fully adjusted models, the association between the single class use and risk for incident severe COVID-19 were statistically significant, suggesting high potential for residual confounding, possibly conferred by indication bias and frailty. Second, the number of quality checks on the data obtained by the Swedish Board of Health and Welfare in 2021 was lower than usual due to the urgency of providing information on the pandemics. Third, since patients with COVID-19 were defined based on hospitalization or death for COVID-19, our results might not be generalizable to COVID-19 patients who were not admitted to the hospital. Although our analyses were adjusted for co-morbidities and pharmacological treatments which might serve as proxies of glycaemic control, data on HbA1c levels were missing thus glycaemic control could not be directly assessed. Finally, patients with T2DM only treated in primary care were not included.

In conclusion, in a nationwide real-world population of patients with T2DM, the use of SGLT2i was associated with a slightly higher risk of incident COVID-19 hospitalization/death, but not with higher 30-day mortality in patients with COVID-19. GLP-1 RA treatment was not significantly associated with a higher risk of COVID-19 hospitalization/death or with increased mortality. The use of DPP-4i was associated with a slightly higher risk of hospitalization/death due to COVID-19 and of 30-day mortality among patients hospitalized with COVID-19. None of the three-drug classes was associated with an increased risk of incident severe COVID-19 or death that exceeded 11%. Thus, these observational results should be interpreted with caution because of the high potential of indication bias, i.e. overall increased vulnerability in patients who are prescribed these drugs.

## Supplementary Material

pvac044_Supplemental_File

## Notes

G.M.C.R. and G.S. are Editors of European Heart Journal—Cardiovascular Pharmacotherapy and were not involved in the peer review process or publication decision.

## Data availability

The data underlying this article are available in the article and in its online supplementary material.

## References

[1] Zhu N , Zhang D, Wang W, Li X, Yang B, Song J et al. A novel coronavirus from patients with Pneumonia in China, 2019. N Engl J Med, 2020;382:727–733. 31978945 10.1056/NEJMoa2001017 PMC7092803

[2] Mantovani A , Byrne CD, Zheng MH, Targher G. Diabetes as a risk factor for greater COVID-19 severity and in-hospital death: a meta-analysis of observational studies. Nutr Metab Cardiovasc Dis 2020;30:1236–1248. 32571616 10.1016/j.numecd.2020.05.014 PMC7258796

[3] Liu D , Wang Y, Zhao B, Lan L, Liu Y, Bao L et al. Overall reduced lymphocyte especially T and B subsets closely related to the poor prognosis and the disease severity in severe patients with COVID-19 and diabetes mellitus. Diabetol Metab Syndr 2021;13:5. 33436069 10.1186/s13098-020-00622-3 PMC7802992

[4] Du F , Liu B, Zhang S. COVID-19: the role of excessive cytokine release and potential ACE2 down-regulation in promoting hypercoagulable state associated with severe illness. J Thromb Thrombolysis 2021;51:313–329. 32676883 10.1007/s11239-020-02224-2 PMC7365308

[5] Katsiki N , Ferrannini E. Anti-inflammatory properties of antidiabetic drugs: a ‘‘promised land’’ in the COVID-19 era? J Diabetes Complications 2020;34:107723. 32900588 10.1016/j.jdiacomp.2020.107723 PMC7448766

[6] Drucker DJ . Coronavirus infections and type 2 diabetes-shared pathways with therapeutic implications. Endocr Rev 2020;41:10.1210/endrev/bnaa011 PMC7184382 32294179

[7] Li Y , Zhang Z, Yang L, Lian X, Xie Y, Li S et al. The MERS-CoV receptor DPP4 as a candidate binding target of the SARS-CoV-2 spike. iScience 2020;23:101160. 32405622 10.1016/j.isci.2020.101160 PMC7219414

[8] Marfella R , Paolisso P, Sardu C, Bergamaschi L, D'Angelo EC, Barbieri M et al. Negative impact of hyperglycaemia on tocilizumab therapy in COVID-19 patients. Diabetes Metab 2020;46:403–405. 32447102 10.1016/j.diabet.2020.05.005 PMC7241396

[9] Yang Y , Cai Z, Zhang J. DPP-4 inhibitors may improve the mortality of coronavirus disease 2019: a meta-analysis. PLoS One 2021;16:e0251916. 34015003 10.1371/journal.pone.0251916 PMC8136680

[10] Abramczyk U , Kuzan A. What every diabetologist should know about SARS-CoV-2: state of knowledge at the beginning of 2021. J Clin Med 2021;10: 1022. 33801468 10.3390/jcm10051022 PMC7958842

[11] Pal R , Bhadada SK. Should anti-diabetic medications be reconsidered amid COVID-19 pandemic? Diabetes Res Clin Pract 2020;163:108146. 32283128 10.1016/j.diabres.2020.108146 PMC7151403

[12] Savarese G , Benson L, Sundstrom J, Lund LH. Association between renin-angiotensin-aldosterone system inhibitor use and COVID-19 hospitalization and death: a 1.4 million patient nationwide registry analysis. Eur J Heart Fail 2021;23:476–485. 33222412 10.1002/ejhf.2060 PMC7753665

[13] Ludvigsson JF , Almqvist C, Bonamy AK, Ljung R, Michaelsson K, Neovius M et al. Registers of the Swedish total population and their use in medical research. Eur J Epidemiol 2016;31:125–136. 26769609 10.1007/s10654-016-0117-y

[14] Zou G . A modified poisson regression approach to prospective studies with binary data. Am J Epidemiol 2004;159:702–706. 15033648 10.1093/aje/kwh090

[15] Barron E , Bakhai C, Kar P, Weaver A, Bradley D, Ismail H et al. Associations of type 1 and type 2 diabetes with COVID-19-related mortality in England: a whole-population study. Lancet Diabetes Endocrinol 2020;8:813–822. 32798472 10.1016/S2213-8587(20)30272-2 PMC7426088

[16] Al Mahmeed W , Al-Rasadi K, Banerjee Y, Ceriello A, Cosentino F, Galia M et al. COvid CAPoIeoS. Promoting a syndemic approach for cardiometabolic disease management during COVID-19: the CAPISCO international expert panel. Front Cardiovasc Med 2021;8:787761. 34977193 10.3389/fcvm.2021.787761 PMC8715947

[17] Bornstein SR , Rubino F, Khunti K, Mingrone G, Hopkins D, Birkenfeld AL et al. Practical recommendations for the management of diabetes in patients with COVID-19. Lancet Diabetes Endocrinol 2020;8:546–550. 32334646 10.1016/S2213-8587(20)30152-2 PMC7180013

[18] Cosentino F , Grant PJ, Aboyans V, Bailey CJ, Ceriello A, Delgado V et al.. 2019 ESC Guidelines on diabetes, pre-diabetes, and cardiovascular diseases developed in collaboration with the EASD. Eur Heart J 2020;41:255–323. 31497854 10.1093/eurheartj/ehz486

[19] Kosiborod MN , Esterline R, Furtado RHM, Oscarsson J, Gasparyan SB, Koch GG et al.. Dapagliflozin in patients with cardiometabolic risk factors hospitalised with COVID-19 (DARE-19): a randomised, double-blind, placebo-controlled, phase 3 trial. Lancet Diabetes Endocrinol 2021;9:586–594. 34302745 10.1016/S2213-8587(21)00180-7 PMC8294807

[20] Filardi T , Morano S. COVID-19: is there a link between the course of infection and pharmacological agents in diabetes? J Endocrinol Invest 2020;43:1053–1060. 32495299 10.1007/s40618-020-01318-1 PMC7268955

[21] Hoffmann M , Kleine-Weber H, Schroeder S, Kruger N, Herrler T, Erichsen S et al. SARS-CoV-2 cell entry depends on ACE2 and TMPRSS2 and is blocked by a clinically proven protease inhibitor. Cell 2020;181:271–280 e278. 32142651 10.1016/j.cell.2020.02.052 PMC7102627

[22] Bonora BM , Avogaro A, Fadini GP. Disentangling conflicting evidence on DPP-4 inhibitors and outcomes of COVID-19: narrative review and meta-analysis. J Endocrinol Invest 2021;44:1379–1386. 33512688 10.1007/s40618-021-01515-6 PMC7845283

[23] Sainsbury C , Wang J, Gokhale K, Acosta-Mena D, Dhalla S, Byne N et al.. Sodium-glucose co-transporter-2 inhibitors and susceptibility to COVID-19: a population-based retrospective cohort study. Diabetes Obes Metab 2021;23:263–269. 32991065 10.1111/dom.14203 PMC7537530

[24] Khunti K , Knighton P, Zaccardi F, Bakhai C, Barron E, Holman N et al. Prescription of glucose-lowering therapies and risk of COVID-19 mortality in people with type 2 diabetes: a nationwide observational study in England. Lancet Diabetes Endocrinol 2021;9:293–303. 33798464 10.1016/S2213-8587(21)00050-4 PMC8009618

[25] Hariyanto TI , Intan D, Hananto JE, Putri C, Kurniawan A. Pre-admission glucagon-like peptide-1 receptor agonist (GLP-1RA) and mortality from coronavirus disease 2019 (COVID-19): a systematic review, meta-analysis, and meta-regression. Diabetes Res Clin Pract 2021;179:109031. 34461139 10.1016/j.diabres.2021.109031 PMC8397482

[26] Solerte SB , D'Addio F, Trevisan R, Lovati E, Rossi A, Pastore I et al. Sitagliptin treatment at the time of hospitalization was associated with reduced mortality in patients with type 2 diabetes and COVID-19: a multicenter, case-control, retrospective, observational study. Diabetes Care 2020;43:2999–3006. 32994187 10.2337/dc20-1521 PMC7770266

[27] Nyland JE , Raja-Khan NT, Bettermann K, Haouzi PA, Leslie DL, Kraschnewski JL et al. Diabetes, drug treatment and mortality in COVID-19: a multinational retrospective cohort study. Diabetes 2021;70: 2903–2916. 34580086 10.2337/db21-0385 PMC8660979

[28] Israelsen SB , Pottegård A, Sandholdt H, Madsbad S, Thomsen RW, Benfield T. Comparable COVID-19 outcomes with current use of GLP-1 receptor agonists, DPP-4 inhibitors or SGLT-2 inhibitors among patients with diabetes who tested positive for SARS-CoV-2. Diabetes Obes Metab 2021;23:1397–1401. 33502076 10.1111/dom.14329 PMC8014019

[29] Pérez-Belmonte LM , Torres-Peña JD, López-Carmona MD, Ayala-Gutiérrez MM, Fuentes-Jiménez F, Huerta LJ et al. Mortality and other adverse outcomes in patients with type 2 diabetes mellitus admitted for COVID-19 in association with glucose-lowering drugs: a nationwide cohort study. BMC Med 2020;18:359. 33190637 10.1186/s12916-020-01832-2 PMC7666969

[30] Roussel R , Darmon P, Pichelin M, Goronflot T, Abouleka Y, Ait Bachir L et al. Use of dipeptidyl peptidase-4 inhibitors and prognosis of COVID-19 in hospitalized patients with type 2 diabetes: a propensity score analysis from the CORONADO study. Diabetes Obes Metab 2021;23:1162–1172. 33528920 10.1111/dom.14324 PMC8013481

[31] Kahkoska AR , Abrahamsen TJ, Alexander GC, Bennett TD, Chute CG, Haendel MA et al. Association between glucagon-like peptide 1 receptor agonist and sodium-glucose cotransporter 2 inhibitor use and COVID-19 outcomes. Diabetes Care 2021;44: 1564–1572. 34135013 10.2337/dc21-0065 PMC8323175

[32] Nishiga M , Wang DW, Han Y, Lewis DB, Wu JC. COVID-19 and cardiovascular disease: from basic mechanisms to clinical perspectives. Nat Rev Cardiol 2020;17:543–558. 32690910 10.1038/s41569-020-0413-9 PMC7370876

