# Corrigendum to: Association between use of novel glucose-lowering drugs and COVID-19 hospitalization and death in patients with type 2 diabetes: A nationwide registry analysis

**DOI:** 10.1093/ehjcvp/pvad011

**Published:** 2023-01-31

This is a corrigendum to: Giulia Ferrannini, Lars H Lund, Lina Benson, Manfredi Rizzo, Wael Almahmeed, Giuseppe M C Rosano, Gianluigi Savarese, Francesco Cosentino, Association between use of novel glucose-lowering drugs and COVID-19 hospitalization and death in patients with type 2 diabetes: a nationwide registry analysis, *European Heart Journal—Cardiovascular Pharmacotherapy*, Volume 9, Issue 1, January 2023, Pages 10–17, https://doi.org/10.1093/ehjcvp/pvac044

In the originally published version of this manuscript the following statement was inadvertently omitted from the ‘Funding’ section:

‘This study was supported by the Italian Ministry of Health (Ricerca Corrente) 20/1819.’

This error has been corrected.

