# Supplementary material for: Association between use of novel glucose-lowering drugs and COVID-19 hospitalization and death in patients with type 2 diabetes: a nationwide registry analysis
Source: Eur Heart J Cardiovasc Pharmacother. 2022 Aug 13;9(1):10–7. doi: 10.1093/ehjcvp/pvac044 (PMC9384777; doi:10.1093/ehjcvp/pvac044)
Supplement: pvac044_Supplemental_File [file pvac044_supplemental_file.docx]

**Supplementary Table S1. Patient selection**

| Exclude patients who died prior to February 1^st^ 2020 | 363,665 |
| --- | --- |
| Exclude patients emigrated prior to December 31^st^, 2019 | 359,704 |
| Exclude patients immigrated post January 1^st^, 2015 | 355,042 |
| Exclude patients with reused or changed PINs | 349,801 |
| Exclude patients < 18 years | 349,455 |
| Exclude patients with missing data for SCB variables included in the models | 344,413 |

NPR: National Patient Registry; PIN: Personal Identification Number; SCB: Statistiska centralbyrån.

**Supplementary Table S2. Variable definitions.**

| **Variable** | **Code** | **Time definition** |
| --- | --- | --- |
| **Comorbidities** | | |
| **Hypertension** | ICD-10: I10-5 | 1997 to February 1^st^ 2020 |
| **Heart failure** | ICD-10: I110, I130, I132, I255, I420, I423, I425-9, I43, I50, J81, K761, R570 | 1997 to February 1^st^ 2020 |
| **Diabetes** | ICD-10: E11 | 1997 to February 1^st^ 2020 |
| **Kidney disease** | ICD-10: N17-9, Z491, Z492. OP: KAS00, KAS10, KAS20, DR012-6, DR020, DR023, DR024, TJA33, TJA35 | 1997 to February 1^st^ 2020 |
| **Ischemic heart disease** | ICD-10: I20-5, Z951, Z955. OP: FNA-H | 1997 to February 1^st^ 2020 |
| **Obesity** | ICD-10: E66 | 1997 to February 1^st^ 2020 |
| **Anemia** | ICD-10: D5, D60-4 | 1997 to February 1^st^ 2020 |
| **Myocardial infarction** | ICD-10: I21, I22, I252 | 1997 to February 1^st^ 2020 |
| **PCI** | OP: FNG | 1997 to February 1^st^ 2020 |
| **CABG** | ICD-10: Z951, Z955. OP: FNA-F, FNH | 1997 to February 1^st^ 2020 |
| **Peripheral artery disease** | ICD-10: I70-3 | 1997 to February 1^st^ 2020 |
| **Atrial fibrillation** | ICD-10: I48 | 1997 to February 1^st^ 2020 |
| **Stroke/TIA** | ICD-10: I60-4, I690-4, G45 | 1997 to February 1^st^ 2020 |
| **Valvular disease** | ICD-10: I05-8, I34-9, Q22, Q230-3, Z952-4 | 1997 to February 1^st^ 2020 |
| **Hyperkalemia** | ICD-10: E875 | 1997 to February 1^st^ 2020 |
| **Hypokalemia** | ICD-10: E876 | 1997 to February 1^st^ 2020 |
| **Dialysis** | ICD-10: Z491, Z492. OP: DR012-6, DR020, DR023, DR024, TJA33, TJA35 | 1997 to February 1^st^ 2020 |
| **COPD** | ICD-10: J40-4 | 1997 to February 1^st^ 2020 |
| **Liver disease** | ICD-10: B18, I85, I864, I982, K70, K710, K711, K713-7, K72-4, K760, K762-9 | 1997 to February 1^st^ 2020 |
| **Dementia** | ICD-10: F00-4 | 1997 to February 1^st^ 2020 |
| **Malignancies (within 3 years)** | ICD-10: C | February 1^st^ 2017 to February 1^st^ 2020 |
| **Musculoskeletal diseases (within 3 years)** | ICD-10: M | February 1^st^ 2017 to February 1^st^ 2020 |
| **Alcohol abuse** | ICD-10: E244, E52, F10, G312, G621, G721, I426, K292, K70, K860, O354, P043, Q860, T51, Z502, Z714- Ecod: Y90, Y91 | 1997 to February 1^st^ 2020 |
| **Major bleeding** | ICD-10: S064-6, I850, I983, K226, K250, K252, K254, K256, K260, K262, K264, K266, K270, K272, K274, K276, K280, K284, K286, K290, K625, K661, K920-2, H431, N02, R04, R58, T810, D629. OP: DR029 | 1997 to February 1^st^ 2020 |
| **Hypoglycaemia** | E16.0, E16.2, E16.1W, E11.0C, E11.6A | February 2^nd^ 2020 to May 15^th^ 2021 |
| **Diabetic ketoacidosis** | E11.1 | February 2^nd^ 2020 to May 15^th^ 2021 |
| **Treatments** | | |
| **SGLT2i** | ATC: A10BK,A10BD1[5-6], A10BD19, A10BD2[0-1], A10BD2[3-5], A10BX09, A10BX11, A10BX12 | September 1^st^ 2019 to February 1^st^ 2020 |
| **GLP-1 RA** | ATC: A10BJ, A10BX04, A10BX07, A10BX10, A10BX1[3-4] | September 1^st^ 2019 to February 1^st^ 2020 |
| **DPP4i** | ATC: A10BH,A10BD0[7-9], A10BD1[0-3], A10BD1[8-9], A10BD2[1-2], A10BD2[4-5] | September 1^st^ 2019 to February 1^st^ 2020 |
| **Insulin** | ATC: A10A | September 1^st^ 2019 to February 1^st^ 2020 |
| **Metformin** | ATC: A10BA02, A10BD0[2-3], A10BD05, A10BD0[7-8], A10BD1[0-1], A10BD1[3-8], A10BD20, A10BD2[2-3], A10BD25 | September 1^st^ 2019 to February 1^st^ 2020 |
| **Other antidiabetic agents** | ATC: A10BB,A10BC01, A10BD0[2-6], A10BD09, A10BD12, A10BD14, A10BD17, A10BF, A10BG, A10BE, A10XA01, A10BX0[2-3], A10BX0[5-6], A10BX08 | September 1^st^ 2019 to February 1^st^ 2020 |
| **ACEi** | ATC: C09A,C09B | September 1^st^ 2019 to February 1^st^ 2020 |
| **ARB** | ATC: C09C, C09D excl.X04 | September 1^st^ 2019 to February 1^st^ 2020 |
| **Diuretics** | ATC: C03 excl.DA, C07B, C07C, C07D, C08GA, C09BA, C09DA, C09DX01 | September 1^st^ 2019 to February 1^st^ 2020 |
| **Beta-blockers** | ATC: C07 | September 1^st^ 2019 to February 1^st^ 2020 |
| **Calcium-channel blockers** | ATC: C08, C07FB, C09BB, C09DB, C09DX01 | September 1^st^ 2019 to February 1^st^ 2020 |
| **MRA** | ATC: C03DA | September 1^st^ 2019 to February 1^st^ 2020 |
| **ARNI** | ATC: C09DX04 | September 1^st^ 2019 to February 1^st^ 2020 |
| **Antiplatelet** | ATC: B01AC | September 1^st^ 2019 to February 1^st^ 2020 |
| **Anticoagulant** | ATC: B01A excl.C | September 1^st^ 2019 to February 1^st^ 2020 |
| **Lipid-lowering agents** | ATC: C10 | September 1^st^ 2019 to February 1^st^ 2020 |
| **Digoxin** | ATC: C01AA05 | September 1^st^ 2019 to February 1^st^ 2020 |
| **Nitrate** | ATC: C01DA | September 1^st^ 2019 to February 1^st^ 2020 |
| **Antiarrhythmic agents** | ATC: C01B | September 1^st^ 2019 to February 1^st^ 2020 |
| **Confirmed COVID-19** | ICD-10: U071 | February 2^nd^ 2020 to May 15^st^ 2021 |

ICD-10 diagnoses refer to hospitalizations since 1997 (when ICD-10 codes were implemented) and out-patient non-primary care visits since 2001 (the latter only for comorbidities). ICD-10 codes were considered in any position for comorbidities and in primary position for the outcomes. Use of treatments was defined as at least one prescription during the time frame indicated above.

**Abbreviations** - ACEi: angiotensin converting enzyme inhibitor; ARB: angiotensin receptor blocker; ARNI: angiotensin receptor neprilysin inhibitor; ATC: anatomical therapeutic chemical; CABG: coronary artery by-pass graft; COPD: chronic obstructive pulmonary disease; COVID-19: coronavirus disease 2019; DPP4i: dipeptidyl peptidase-4 inhibitors; GLP-1 RA: glucagon-like peptide-1 receptor agonists; ICD: international classification of diseases; MRA: mineralocorticoid receptor antagonist; PCI: percutaneous coronary intervention; SGLT2i: sodium-glucose cotransporter 2 inhibitors; TIA: transient ischemic attack.

**Supplementary Table S3.** Baseline characteristics of patients with type 2 diabetes according to the use of sodium-glucose cotransporter 2 inhibitors, glucagon-like peptide-1 receptor agonists and dipeptidyl peptidase-4 inhibitors.

Categorical variables are presented with n (%) and continuous variables with median [q1-q3].

| VARIABLE | SGLT2i No | SGLT2i Yes | SMD | GLP1-RA No | GLP1-RA Yes | SMD | DPP-4i No | DPP-4i Yes | SMD |
| --- | --- | --- | --- | --- | --- | --- | --- | --- | --- |
| n | 305241 | 39172 |  | 310123 | 34290 |  | 291369 | 53044 |  |
| Covid period^†^ |  |  | 0.177 |  |  | 0.215 |  |  | 0.066 |
| Feb-Jun 2020 | 2877 (33.6) | 267 (27.7) |  | 2882 (33.4) | 262 (28.9) |  | 2645 (33.5) | 499 (30.4) |  |
| Jan-May 2021 | 2815 (32.8) | 395 (41.0) |  | 2820 (32.7) | 390 (43.0) |  | 2643 (33.5) | 567 (34.6) |  |
| Jul-Dec 2020 | 2883 (33.6) | 301 (31.3) |  | 2929 (33.9) | 255 (28.1) |  | 2611 (33.1) | 573 (35.0) |  |
| Age* | 72.0  [63.0, 79.0] | 66.0  [59.0, 73.0] | 0.433 | 72.0  [63.0, 79.0] | 66.0  [57.0, 73.0] | 0.502 | 71.0  [62.0, 78.0] | 73.0  [65.0, 79.0] | 0.155 |
| male sex* | 172002 (56.3) | 26461 (67.6) | 0.232 | 178181 (57.5) | 20282 (59.1) | 0.034 | 167018 (57.3) | 31445 (59.3) | 0.040 |
| Children (yes)* | 249440 (81.7) | 31415 (80.2) | 0.039 | 253736 (81.8) | 27119 (79.1) | 0.069 | 237223 (81.4) | 43632 (82.3) | 0.022 |
| Country of birth* |  |  | 0.087 |  |  | 0.016 |  |  | 0.014 |
| Sweden | 247641 (81.1) | 30525 (77.9) |  | 250301 (80.7) | 27865 (81.3) |  | 235561 (80.8) | 42605  (80.3) |  |
| Europe | 33659 (11.0) | 4722 (12.1) |  | 34605 (11.2) | 3776 (11.0) |  | 32375 (11.1) | 6006 (11.3) |  |
| Other | 23941 (7.8) | 3925 (10.0) |  | 25217 (8.1) | 2649 (7.7) |  | 23433 (8.0) | 4433 (8.4) |  |
| Income (categories)* ^‡^ |  |  | 0.235 |  |  | 0.242 |  |  | 0.050 |
| Low | 102413 (33.6) | 11001 (28.1) |  | 103955 (33.5) | 9459 (27.6) |  | 95561 (32.8) | 17853  (33.7) |  |
| Medium | 102961 (33.7) | 10914 (27.9) |  | 104319 (33.6) | 9556 (27.9) |  | 95686 (32.8) | 18189 (34.3) |  |
| High | 99867 (32.7) | 17257 (44.1) |  | 101849 (32.8) | 15275 (44.5) |  | 100122 (34.4) | 17002  (32.1) |  |
| Education* |  |  | 0.112 |  |  | 0.161 |  |  | 0.067 |
| Compulsory | 104399 (34.2) | 11439 (29.2) |  | 106570 (34.4) | 9268 (27.0) |  | 96618 (33.2) | 19220 (36.2) |  |
| Secondary school | 136771 (44.8) | 19371 (49.5) |  | 138801 (44.8) | 17341 (50.6) |  | 132737 (45.6) | 23405  (44.1) |  |
| University | 64071 (21.0) | 8362 (21.3) |  | 64752 (20.9) | 7681 (22.4) |  | 62014 (21.3) | 10419 (19.6) |  |
| Living alone (yes)* | 144594 (47.4) | 16212 (41.4) | 0.121 | 146439 (47.2) | 14367 (41.9) | 0.107 | 136344 (46.8) | 24462 (46.1) | 0.014 |
| Region Stockholm* | 61992 (20.3) | 5577 (14.2) | 0.161 | 61047 (19.7) | 6522 (19.0) | 0.017 | 60413 (20.7) | 7156 (13.5) | 0.193 |
| Comorbidities | | | | | | | | | |
| Atrial fibrillation* | 55315 (18.1) | 5765 (14.7) | 0.092 | 56307 (18.2) | 4773 (13.9) | 0.116 | 51080 (17.5) | 10000 (18.9) | 0.034 |
| Alcohol overconsumption* | 17988 (5.9) | 1874 (4.8) | 0.049 | 18274 (5.9) | 1588 (4.6) | 0.057 | 17567 (6.0) | 2295 (4.3) | 0.077 |
| Anemia* | 45069 (14.8) | 3636 (9.3) | 0.169 | 44737 (14.4) | 3968 (11.6) | 0.085 | 40934 (14.0) | 7771 (14.7) | 0.017 |
| Bleeding event* | 73561 (24.1) | 7076 (18.1) | 0.148 | 74047 (23.9) | 6590 (19.2) | 0.113 | 68668 (23.6) | 11969 (22.6) | 0.024 |
| CABG* | 44760 (14.7) | 8577 (21.9) | 0.188 | 48000 (15.5) | 5337 (15.6) | 0.002 | 44787 (15.4) | 8550 (16.1) | 0.021 |
| Cancer in the last 3 years* | 38118 (12.5) | 3234 (8.3) | 0.139 | 38456 (12.4) | 2896 (8.4) | 0.130 | 34683 (11.9) | 6669 (12.6) | 0.020 |
| COPD* | 23421 (7.7) | 2267 (5.8) | 0.075 | 23458 (7.6) | 2230 (6.5) | 0.041 | 21707 (7.5) | 3981 (7.5) | 0.002 |
| Dementia* | 10713 (3.5) | 406 (1.0) | 0.167 | 10785 (3.5) | 334 (1.0) | 0.170 | 9473 (3.3) | 1646 (3.1) | 0.008 |
| Dialysis* | 4352 (1.4) | 153 (0.4) | 0.109 | 4196 (1.4) | 309 (0.9) | 0.043 | 3737 (1.3) | 768 (1.4) | 0.014 |
| Heart failure* | 44595 (14.6) | 5286 (13.5) | 0.032 | 45462 (14.7) | 4419 (12.9) | 0.051 | 41379 (14.2) | 8502 (16.0) | 0.051 |
| Hyperkalemia* | 4539 (1.5) | 295 (0.8) | 0.070 | 4403 (1.4) | 431 (1.3) | 0.014 | 3943 (1.4) | 891 (1.7) | 0.027 |
| Hypertension* | 214204 (70.2) | 27177 (69.4) | 0.017 | 216942 (70.0) | 24439 (71.3) | 0.029 | 202744 (69.6) | 38637 (72.8) | 0.072 |
| Hypokalemia* | 7996 (2.6) | 587 (1.5) | 0.079 | 7944 (2.6) | 639 (1.9) | 0.047 | 7400 (2.5) | 1183 (2.2) | 0.020 |
| ICD/CRT* | 3499 (1.1) | 710 (1.8) | 0.055 | 3688 (1.2) | 521 (1.5) | 0.029 | 3554 (1.2) | 655 (1.2) | 0.001 |
| Ischaemic heart disease* | 80260 (26.3) | 13218 (33.7) | 0.163 | 84600 (27.3) | 8878 (25.9) | 0.031 | 78691 (27.0) | 14787 (27.9) | 0.019 |
| Liver disease* | 12371 (4.1) | 1581 (4.0) | 0.001 | 12404 (4.0) | 1548 (4.5) | 0.025 | 12062 (4.1) | 1890 (3.6) | 0.030 |
| Previous myocardial infarction* | 49546 (16.2) | 8757 (22.4) | 0.156 | 52752 (17.0) | 5551 (16.2) | 0.022 | 49222 (16.9) | 9081 (17.1) | 0.006 |
| Musculoskeletal disease* | 93713 (30.7) | 10791 (27.5) | 0.069 | 93759 (30.2) | 10745 (31.3) | 0.024 | 88551 (30.4) | 15953 (30.1) | 0.007 |
| Obesity* | 45471 (14.9) | 8316 (21.2) | 0.165 | 43150 (13.9) | 10637 (31.0) | 0.419 | 46710 (16.0) | 7077 (13.3) | 0.076 |
| PCI* | 37155 (12.2) | 7740 (19.8) | 0.208 | 40155 (12.9) | 4740 (13.8) | 0.026 | 37791 (13.0) | 7104 (13.4) | 0.012 |
| Peripheral artery disease* | 24233 (7.9) | 2667 (6.8) | 0.043 | 24714 (8.0) | 2186 (6.4) | 0.062 | 22656 (7.8) | 4244 (8.0) | 0.008 |
| Renal disease* | 34401 (11.3) | 1648 (4.2) | 0.267 | 32427 (10.5) | 3622 (10.6) | 0.003 | 28318 (9.7) | 7731 (14.6) | 0.149 |
| Previous Stroke/TIA* | 50345 (16.5) | 5020 (12.8) | 0.104 | 51456 (16.6) | 3909 (11.4) | 0.150 | 46668 (16.0) | 8697 (16.4) | 0.010 |
| Valvular disease* | 19550 (6.4) | 1994 (5.1) | 0.056 | 19974 (6.4) | 1570 (4.6) | 0.082 | 18117 (6.2) | 3427 (6.5) | 0.010 |
| Pharmacological therapy | | | | | | | | | |
| Antiarrhythmic* | 2076 (0.7) | 348 (0.9) | 0.024 | 2157 (0.7) | 267 (0.8) | 0.010 | 2036 (0.7) | 388 (0.7) | 0.004 |
| anticoagulant* | 55968 (18.3) | 6132 (15.7) | 0.071 | 56868 (18.3) | 5232 (15.3) | 0.082 | 51876 (17.8) | 10224 (19.3) | 0.038 |
| Antiplatlet* | 100067 (32.8) | 15653 (40.0) | 0.150 | 104173 (33.6) | 11547 (33.7) | 0.002 | 96678 (33.2) | 19042 (35.9) | 0.057 |
| Beta blockers * | 138010 (45.2) | 19816 (50.6) | 0.108 | 141082 (45.5) | 16744 (48.8) | 0.067 | 130696 (44.9) | 27130 (51.1) | 0.126 |
| Calcium channel blockers* | 105030 (34.4) | 13662 (34.9) | 0.010 | 106007 (34.2) | 12685 (37.0) | 0.059 | 98173 (33.7) | 20519 (38.7) | 0.104 |
| Digoxin* | 6282 (2.1) | 927 (2.4) | 0.021 | 6538 (2.1) | 671 (2.0) | 0.011 | 5894 (2.0) | 1315 (2.5) | 0.031 |
| Diuretics* | 111387 (36.5) | 13088 (33.4) | 0.065 | 110467 (35.6) | 14008 (40.9) | 0.108 | 102751 (35.3) | 21724 (41.0) | 0.117 |
| dpp-4i* | 44593 (14.6) | 8451 (21.6) | 0.182 | 50351 (16.2) | 2693 (7.9) | 0.260 | 0 (0.0) | 53044 (100.0) | - |
| glp1-ra* | 25444 (8.3) | 8846 (22.6) | 0.402 | 0 (0.0) | 34290 (100.0) | - | 31597 (10.8) | 2693 (5.1) | 0.214 |
| Insulin* | 107498 (35.2) | 15932 (40.7) | 0.113 | 105239 (33.9) | 18191 (53.1) | 0.393 | 105761 (36.3) | 17669 (33.3) | 0.063 |
| Lipid-lowering* | 185554 (60.8) | 30415 (77.6) | 0.371 | 190623 (61.5) | 25346 (73.9) | 0.269 | 177739 (61.0) | 38230 (72.1) | 0.236 |
| Metformin* | 154796 (50.7) | 29999 (76.6) | 0.558 | 162091 (52.3) | 22704 (66.2) | 0.287 | 151956 (52.2) | 32839 (61.9) | 0.198 |
| mra* | 21172 (6.9) | 3423 (8.7) | 0.067 | 21620 (7.0) | 2975 (8.7) | 0.064 | 20479 (7.0) | 4116 (7.8) | 0.028 |
| Nitrates* | 24170 (7.9) | 4104 (10.5) | 0.089 | 25432 (8.2) | 2842 (8.3) | 0.003 | 23594 (8.1) | 4680 (8.8) | 0.026 |
| Other oral antidiabetic* | 26664 (8.7) | 5679 (14.5) | 0.181 | 28289 (9.1) | 4054 (11.8) | 0.088 | 23879 (8.2) | 8464 (16.0) | 0.240 |
| rasi/arni* | 189049 (61.9) | 28361 (72.4) | 0.224 | 192892 (62.2) | 24518 (71.5) | 0.199 | 180954 (62.1) | 36456 (68.7) | 0.140 |
| sglt2i | 0 (0.0) | 39172 (100.0) | - | 30326 (9.8) | 8846 (25.8) | 0.428 | 30721 (10.5) | 8451 (15.9) | 0.160 |

**Abbreviations**

CABG: coronary artery bypass graft; COPD: chronic obstructive pulmonary disease; DPP-4i: dipeptidyl peptidase-4 inhibitors; GLP-1 RA: glucagon-like peptide-1 receptor agonists; ICD/CRT: implantable cardioverter defibrillator/cardiac resynchronization therapy; MRA: mineralocorticoid receptor antagonists; PCI: percutaneous coronary interventions; RASi/ARNi: renin-angiotensin system inhibitors/ angiotensin receptor-neprilysin inhibitors; SGLT2i: Sodium-glucose cotransporter 2 inhibitors; SMD stardardised mean difference; TIA: transitory ischaemic attack.

**^†^** Included in the cox regression/propensity score models for outcome death as a continuous variable for each month (Jan 2021 is month 13 ect).

* Included in the logistic/cox regression/propensity score models.

^‡^ Categorized according to tertiles.

**Supplementary Table S4.**

Baseline characteristics of patients hospitalized for COVID-19 according to the use of the three different drug classes.

| VARIABLE | SGLT2i No | SGLT2i Yes | SMD | GLP1-RA No | GLP1-RA Yes | SMD | DPP-4i No | DPP-4i Yes | SMD |
| --- | --- | --- | --- | --- | --- | --- | --- | --- | --- |
| n | 8575 | 963 |  | 8631 | 907 |  | 7899 | 1639 |  |
| Covid period^†^ |  |  | 0.177 |  |  | 0.215 |  |  | 0.066 |
| Feb-Jun 2020 | 2877 (33.6) | 267 (27.7) |  | 2882 (33.4) | 262 (28.9) |  | 2645 (33.5) | 499 (30.4) |  |
| Jan-May 2021 | 2815 (32.8) | 395 (41.0) |  | 2820 (32.7) | 390 (43.0) |  | 2643 (33.5) | 567 (34.6) |  |
| Jul-Dec 2020 | 2883 (33.6) | 301 (31.3) |  | 2929 (33.9) | 255 (28.1) |  | 2611 (33.1) | 573 (35.0) |  |
| Age* | 76.0 [67.0, 83.0] | 67.0 [59.0, 73.5] | 0.705 | 76.0 [67.0, 83.0] | 66.0 [58.0, 74.0] | 0.742 | 75.0 [65.0, 82.0] | 75.0 [67.0, 82.0] |  |
| male sex* | 5111 (59.6) | 705 (73.2) | 0.291 | 5218 (60.5) | 598 (65.9) | 0.114 | 4786 (60.6) | 1030 (62.8) | 0.046 |
| Children (yes)* | 7144 (83.3) | 789 (81.9) | 0.036 | 7183 (83.2) | 750 (82.7) | 0.014 | 6580 (83.3) | 1353 (82.6) | 0.020 |
| Country of birth* |  |  | 0.166 |  |  | 0.042 |  |  | 0.017 |
| Sweden | 6044 (70.5) | 610 (63.3) |  | 6037 (69.9) | 617 (68.0) |  | 5500 (69.6) | 1154 (70.4) |  |
| Europe | 1403 (16.4) | 175 (18.2) |  | 1420 (16.5) | 158 (17.4) |  | 1314 (16.6) | 264 (16.1) |  |
| Other | 1128 (13.2) | 178 (18.5) |  | 1174 (13.6) | 132 (14.6) |  | 1085 (13.7) | 221 (13.5) |  |
| Income (categories)* ^‡^ |  |  | 0.323 |  |  | 0.326 |  |  | 0.030 |
| Low | 3177 (37.0) | 326 (33.9) |  | 3213 (37.2) | 290 (32.0) |  | 2883 (36.5) | 620 (37.8) |  |
| Medium | 3284 (38.3) | 264 (27.4) |  | 3289 (38.1) | 259 (28.6) |  | 2943 (37.3) | 605 (36.9) |  |
| High | 2114 (24.7) | 373 (38.7) |  | 2129 (24.7) | 358 (39.5) |  | 2073 (26.2) | 414 (25.3) |  |
| Education* |  |  | 0.088 |  |  | 0.228 |  |  | 0.031 |
| Compulsory | 3505 (40.9) | 354 (36.8) |  | 3578 (41.5) | 281 (31.0) |  | 3183 (40.3) | 676 (41.2) |  |
| Secondary school | 3538 (41.3) | 434 (45.1) |  | 3514 (40.7) | 458 (50.5) |  | 3310 (41.9) | 662 (40.4) |  |
| University | 1532 (17.9) | 175 (18.2) |  | 1539 (17.8) | 168 (18.5) |  | 1406 (17.8) | 301 (18.4) |  |
| Living alone (yes)* | 4416 (51.5) | 384 (39.9) | 0.235 | 4427 (51.3) | 373 (41.1) | 0.205 | 3988 (50.5) | 812 (49.5) | 0.019 |
| Region Stockholm* | 2856 (33.3) | 240 (24.9) | 0.185 | 2804 (32.5) | 292 (32.2) | 0.006 | 2719 (34.4) | 377 (23.0) | 0.254 |
| Comorbidities | | | | | | | | | |
| Atrial fibrillation* | 2221 (25.9) | 179 (18.6) | 0.177 | 2227 (25.8) | 173 (19.1) | 0.162 | 1966 (24.9) | 434 (26.5) | 0.036 |
| Alcohol overconsumption* | 480 (5.6) | 43 (4.5) | 0.052 | 484 (5.6) | 39 (4.3) | 0.060 | 460 (5.8) | 63 (3.8) | 0.092 |
| Anemia* | 1972 (23.0) | 134 (13.9) | 0.236 | 1963 (22.7) | 143 (15.8) | 0.178 | 1742 (22.1) | 364 (22.2) | 0.004 |
| Bleeding event* | 2936 (34.2) | 232 (24.1) | 0.225 | 2924 (33.9) | 244 (26.9) | 0.152 | 2634 (33.3) | 534 (32.6) | 0.016 |
| CABG* | 1559 (18.2) | 247 (25.6) | 0.181 | 1641 (19.0) | 165 (18.2) | 0.021 | 1486 (18.8) | 320 (19.5) | 0.018 |
| Cancer in the last 3 years* | 1325 (15.5) | 87 (9.0) | 0.197 | 1320 (15.3) | 92 (10.1) | 0.155 | 1171 (14.8) | 241 (14.7) | 0.003 |
| COPD* | 1170 (13.6) | 94 (9.8) | 0.121 | 1162 (13.5) | 102 (11.2) | 0.067 | 1048 (13.3) | 216 (13.2) | 0.003 |
| Dementia* | 742 (8.7) | 19 (2.0) | 0.301 | 737 (8.5) | 24 (2.6) | 0.259 | 647 (8.2) | 114 (7.0) | 0.047 |
| Dialysis* | 310 (3.6) | 9 (0.9) | 0.181 | 304 (3.5) | 15 (1.7) | 0.118 | 269 (3.4) | 50 (3.1) | 0.020 |
| Heart failure* | 2198 (25.6) | 205 (21.3) | 0.103 | 2219 (25.7) | 184 (20.3) | 0.129 | 1964 (24.9) | 439 (26.8) | 0.044 |
| Hyperkalemia* | 272 (3.2) | 20 (2.1) | 0.069 | 270 (3.1) | 22 (2.4) | 0.043 | 249 (3.2) | 43 (2.6) | 0.032 |
| Hypertension* | 6745 (78.7) | 712 (73.9) | 0.111 | 6760 (78.3) | 697 (76.8) | 0.035 | 6152 (77.9) | 1305 (79.6) | 0.043 |
| Hypokalemia* | 387 (4.5) | 27 (2.8) | 0.091 | 387 (4.5) | 27 (3.0) | 0.080 | 346 (4.4) | 68 (4.1) | 0.011 |
| ICD/CRT* | 142 (1.7) | 31 (3.2) | 0.101 | 151 (1.7) | 22 (2.4) | 0.047 | 140 (1.8) | 33 (2.0) | 0.018 |
| Ischaemic heart disease* | 2942 (34.3) | 390 (40.5) | 0.128 | 3040 (35.2) | 292 (32.2) | 0.064 | 2733 (34.6) | 599 (36.5) | 0.041 |
| Liver disease* | 422 (4.9) | 52 (5.4) | 0.022 | 424 (4.9) | 50 (5.5) | 0.027 | 408 (5.2) | 66 (4.0) | 0.054 |
| Previous myocardial infarction* | 1916 (22.3) | 262 (27.2) | 0.113 | 1990 (23.1) | 188 (20.7) | 0.056 | 1783 (22.6) | 395 (24.1) | 0.036 |
| Musculoskeletal disease* | 3246 (37.9) | 337 (35.0) | 0.059 | 3212 (37.2) | 371 (40.9) | 0.076 | 2986 (37.8) | 597 (36.4) | 0.029 |
| Obesity* | 1489 (17.4) | 254 (26.4) | 0.219 | 1390 (16.1) | 353 (38.9) | 0.528 | 1479 (18.7) | 264 (16.1) | 0.069 |
| PCI* | 1243 (14.5) | 229 (23.8) | 0.238 | 1338 (15.5) | 134 (14.8) | 0.020 | 1198 (15.2) | 274 (16.7) | 0.042 |
| Peripheral artery disease* | 1059 (12.3) | 96 (10.0) | 0.076 | 1059 (12.3) | 96 (10.6) | 0.053 | 947 (12.0) | 208 (12.7) | 0.021 |
| Renal disease* | 1896 (22.1) | 85 (8.8) | 0.374 | 1812 (21.0) | 169 (18.6) | 0.059 | 1539 (19.5) | 442 (27.0) | 0.178 |
| Previous Stroke/TIA* | 2096 (24.4) | 156 (16.2) | 0.206 | 2102 (24.4) | 150 (16.5) | 0.195 | 1866 (23.6) | 386 (23.6) | 0.002 |
| Valvular disease* | 755 (8.8) | 65 (6.7) | 0.077 | 766 (8.9) | 54 (6.0) | 0.112 | 656 (8.3) | 164 (10.0) | 0.059 |
| Pharmacological therapy | | | | | | | | | |
| Antiarrhythmic* | 65 (0.8) | 15 (1.6) | 0.075 | 72 (0.8) | 8 (0.9) | 0.005 | 66 (0.8) | 14 (0.9) | 0.002 |
| anticoagulant* | 2135 (24.9) | 203 (21.1) | 0.091 | 2161 (25.0) | 177 (19.5) | 0.133 | 1898 (24.0) | 440 (26.8) | 0.065 |
| Antiplatlet* | 3169 (37.0) | 396 (41.1) | 0.085 | 3233 (37.5) | 332 (36.6) | 0.018 | 2930 (37.1) | 635 (38.7) | 0.034 |
| Beta blockers * | 4444 (51.8) | 526 (54.6) | 0.056 | 4498 (52.1) | 472 (52.0) | 0.001 | 4037 (51.1) | 933 (56.9) | 0.117 |
| Calcium channel blockers* | 2997 (35.0) | 342 (35.5) | 0.012 | 3008 (34.9) | 331 (36.5) | 0.034 | 2700 (34.2) | 639 (39.0) | 0.100 |
| Digoxin* | 226 (2.6) | 40 (4.2) | 0.084 | 245 (2.8) | 21 (2.3) | 0.033 | 210 (2.7) | 56 (3.4) | 0.044 |
| Diuretics* | 3883 (45.3) | 375 (38.9) | 0.129 | 3821 (44.3) | 437 (48.2) | 0.078 | 3446 (43.6) | 812 (49.5) | 0.119 |
| dpp-4i* | 1426 (16.6) | 213 (22.1) | 0.139 | 1541 (17.9) | 98 (10.8) | 0.202 | 0 (0.0) | 1639 (100.0) | - |
| glp1-ra* | 683 (8.0) | 224 (23.3) | 0.431 | 0 (0.0) | 907 (100.0) | - | 809 (10.2) | 98 (6.0) | 0.157 |
| Insulin* | 3563 (41.6) | 449 (46.6) | 0.102 | 3484 (40.4) | 528 (58.2) | 0.363 | 3346 (42.4) | 666 (40.6) | 0.035 |
| Lipid-lowering* | 5070 (59.1) | 737 (76.5) | 0.379 | 5137 (59.5) | 670 (73.9) | 0.308 | 4679 (59.2) | 1128 (68.8) | 0.201 |
| Metformin* | 3727 (43.5) | 700 (72.7) | 0.620 | 3863 (44.8) | 564 (62.2) | 0.355 | 3592 (45.5) | 835 (50.9) | 0.110 |
| mra* | 769 (9.0) | 120 (12.5) | 0.113 | 787 (9.1) | 102 (11.2) | 0.070 | 716 (9.1) | 173 (10.6) | 0.050 |
| Nitrates* | 1033 (12.0) | 152 (15.8) | 0.108 | 1071 (12.4) | 114 (12.6) | 0.005 | 955 (12.1) | 230 (14.0) | 0.058 |
| Other oral antidiabetic* | 789 (9.2) | 152 (15.8) | 0.200 | 828 (9.6) | 113 (12.5) | 0.092 | 660 (8.4) | 281 (17.1) | 0.266 |
| rasi/arni* | 5152 (60.1) | 701 (72.8) | 0.272 | 5206 (60.3) | 647 (71.3) | 0.234 | 4794 (60.7) | 1059 (64.6) | 0.081 |
| sglt2i | 0 (0.0) | 963 (100.0) | - | 739 (8.6) | 224 (24.7) | 0.444 | 750 (9.5) | 213 (13.0) | 0.111 |

**Abbreviations**

CABG: coronary artery bypass graft; COPD: chronic obstructive pulmonary disease; DPP-4i: dipeptidyl peptidase-4 inhibitors; GLP-1 RA: glucagon-like peptide-1 receptor agonists; ICD/CRT: implantable cardioverter defibrillator/cardiac resynchronization therapy; MRA: mineralocorticoid receptor antagonists; PCI: percutaneous coronary interventions; RASi/ARNi: renin-angiotensin system inhibitors/ angiotensin receptor-neprilysin inhibitors; SGLT2i: Sodium-glucose cotransporter 2 inhibitors; SMD stardardised mean difference; TIA: transitory ischaemic attack.

**^†^** Included in the cox regression/propensity score models for outcome death as a continuous variable for each month (Jan 2021 is month 13 ect).

* Included in the logistic/cox regression/propensity score models.

^‡^ Categorized according to tertiles.

**Supplementary Table S5.**

Hospitalizations for hypoglycaemia after hospitalization for COVID-19 according to the use of the three different drug classes.

|  | Matched | | | | All patients | | | |
| --- | --- | --- | --- | --- | --- | --- | --- | --- |
| On therapy | No | | Yes | | No | | Yes | |
|  | N | IR/100 py (95% CI) | N | IR/100 py (95% CI) | N | IR/100 py (95% CI) | N | IR/100 py (95% CI) |
| SGLT2i | 2 | 5 (1-19) | 2 | 5 (1-19) | 7 | 2 (1-5) | 2 | 5 (1-19) |
| GLP1- RA | 0 | 0 (0-11) | 1 | 3 (0-16) | 8 | 3 (1-5) | 1 | 3 (0-15) |
| DPP-4i | 0 | 0 (0-7) | 5 | 9 (3-22) | 4 | 1 (0-4) | 5 | 9 (3-22) |

**Abbreviations**

CI: confidence interval; DPP-4i: dipeptidyl peptidase-4 inhibitors; GLP-1 RA: glucagon-like peptide-1 receptor agonists; IR: incidence rate; py: person-year; SGLT2i: Sodium-glucose cotransporter 2 inhibitors
